# Dysfunctional suction dynamics in newborns with ankyloglossia

**DOI:** 10.1590/2317-1782/20232023054

**Published:** 2023-11-10

**Authors:** Bruna Mendes Lourenço Cunha, Erika Baptista Luiz Badarane, Paulo Vitor Melo Sousa, Kauê Machado Costa, Manoel da Silva

**Affiliations:** 1 Laboratório de Prototipagem Assistiva, Programa de Pós-graduação em Neurociências e Biologia Celular, Instituto de Ciências Biológicas, Universidade Federal do Pará - UFPA - Belém (PA), Brasil.; 2 National Institute on Drug Abuse Intramural Research Program, National Institutes of Health - Baltimore, MD, United States of America.

**Keywords:** Ankyloglossia, Sucking Behavior, Newborn, Breast Feeding, Lingual Frenum, Anquiloglossia, Comportamento de Sucção, Recém-Nascido, Aleitamento Materno, Freio Lingual

## Abstract

**Purpose:**

Compare infant suction in babies with and without ankyloglossia using a microprocessor-controlled pressure sensor coupled to a pacifier.

**Methods:**

Fifty-five infants from 0 to 2 months of age underwent clinical examination for ankyloglossia, after which they were offered a silicone pacifier connected to the pressure acquisition

device and suction activity was recorded. Thus, we extracted the frequency of sucks within a burst, the average suck duration, the burst duration, the number of sucks per burst, the maximum amplitude of sucks per burst and the inter-burst interval.

**Results:**

The key difference in newborns with ankyloglossia in relation to control was that they perform longer bursts of suction activity.

**Conclusion:**

The longer burst durations are likely a compensatory strategy and may underlie the pain reported by mothers during breastfeeding. We therefore propose a method for objectively quantifying some parameters of infant suction capacity and demonstrate its use in assisting the evaluation of ankyloglossia.

## RESUMO

**Objetivo:**

Comparar a sucção infantil em bebês com e sem anquiloglossia usando um sensor de pressão controlado por microprocessador acoplado a uma chupeta.

**Método:**

Cinquenta e cinco lactentes de 0 a 2 meses de idade foram submetidos ao exame clínico de anquiloglossia, em seguida foi oferecido uma chupeta de silicone conectada ao dispositivo de aquisição de pressão e a atividade de sucção foi registrada. Assim, obtivemos dados sobre a frequência de sucções dentro de um período de sucções, a duração média da sucção, a duração da rajada, o número de sucções por rajada, a amplitude máxima das sucções por rajada e o intervalo entre rajadas. O teste t não pareado foi utilizado para comparações entre os grupos.

**Resultados:**

A principal diferença dos recém-nascidos com anquiloglossia em relação aos do grupo controle é que eles realizam rajadas mais longas durante a atividade de sucção.

**Conclusão:**

A duração mais longa das rajadas é provavelmente uma estratégia compensatória e pode estar por trás da dor relatada pelas mães durante a amamentação. Portanto, propomos um método para quantificar objetivamente alguns parâmetros da sucção infantil e demonstramos seu uso para auxiliar na avaliação da anquiloglossia.

## INTRODUCTION

It is recommended that breastfeeding starts in the delivery room and that it is exclusive and on demand (the baby breastfeeds the amount he wants, when he wants) until the 6^th^ month and if extended up to 2 years or more^(1)^. Early weaning, i.e., the interruption of breastfeeding prior to those setpoints, can lead to malnutrition and myofunctional impairments^(2)^. Early weaning can be triggered by factors such as nipple-areolar complex trauma, socioeconomic and intellectual issues, the mother's return to professional life, use of pacifiers and baby formula, insufficient milk supply, and, most notably, the presence of ankyloglossia^(3)^. Ankyloglossia, also known as tongue-tie, occurs when a part of the tissue in the sublingual region that should have undergone apoptosis during embryonic development remains, restricting lingual movement^(4)^. Children with ankyloglossia are often unable to properly latch to the nipple-areolar complex, which impairs breastfeeding and can lead to suboptimal weight gains^(5)^.

There are several indicators of ankyloglossia, which include difficulties in latching, inefficient and longer lasting feeding bouts, development of a clicking sound when the child is feeding, and gastric reflux, as well as mastitis and reduction in milk supply for the mother^(6,7)^. These infants cannot properly extend their tongues, and have difficulties moving their tongue from side to side^(8)^. This is critical, as sucking requires wavelike movements of the tongue, in addition to helping to seal the mouth to the nipple, and in the oral preparatory phase of swallowing the milk bolus is centralized and propelled by the tongue^(9,10)^. While there can be asymptomatic ankyloglossia, often from the very beginning of breastfeeding mothers report problems like pain and fissure in the nipple-areolar complex, improper latching, and low milk supply^(11)^.

To date, studies on the relationship between ankyloglossia, breastfeeding difficulties, and the possibility of early weaning have relied primarily on subjective assessments of parental complaints^(3,7,12)^. This is problematic, because frenotomy (the surgical division of the tongue shortened frenulum) is often recommended (or not) without a functional indication of the impairments or potential benefits for the individual infant patient. Since these decisions are based almost exclusively on the provider’s subjective evaluation and experience, there can be a wide range of opinions about what steps to take to mitigate ankyloglossia^(3,6,13,14)^.

Given the potential health consequences of undiagnosed ankyloglossia, and the current reliance on subjective reporting, there is a need for an objective method for early detection and evaluation of this condition. To address this, we developed an inexpensive device capable of measuring an infant’s suction dynamics during non-nutritive suction in order to compare the suction of babies with and without ankyloglossia. We hypothesized that there would be differences in the sucking dynamics of these babies due to maternal reports. Quantifying this behavior could be a first step towards developing a quantitative method for deciding when to recommend frenotomy.

## METHODS

### Participants

This study was carried out at the Santa Casa de Misericórdia do Pará Foundation (FSCMP), with the approval of the ethics committee of the Institute of Health Sciences of the Federal University of Pará (protocol #48449615.1.0000.5171). All research was performed in accordance with relevant guidelines and regulations specific for human research, including the Declaration of Helsinki. Informed consent was obtained in writing from the legal guardians of all the subjects tested in this study. Subjects were babies from 0 to 2 months of age participated, who were exclusively breastfeeding or not. All infants were admitted through the hospital’s general procedure and were in convention post-delivery rooms. We excluded premature babies and newborns with genetic syndromes, craniofacial malformations, breathing disorders or otherwise clinically unstable. Obeying these prerequisites, we obtained a sample of 55 babies.

Each subject experienced the following sequence of procedures: medical record evaluation, anamnesis, physical examination, and recordings with our new suction-measuring device. After anamnesis and evaluation, we observed that the sample contained 19 babies with ankyloglossia (control group = 36).

### Procedures

In evaluating medical records, data were collected to identify the mother, prenatal care, childbirth, infant and hospitalization, and possible complications. This information was intended to identify babies who had any of the exclusion criteria. We selected full-term newborns (gestational age between 38 and 41 weeks) who did not present complications at birth, complications after birth and did not have any identified syndromes. In the anamnesis, data collected in the medical record was confirmed, and also reports about breastfeeding. We asked mothers about their pain while breastfeeding and the perceived duration of breastfeeding, in order to reveal whether the differences between the groups could have an impact on the mothers' breastfeeding routine, and to corroborate our hypothesis that measuring non-nutritive suction could be informative as to nutritive suction patterns and the clinical effects of ankyloglossia. The physical examination phase was performed by an otolaryngologist using the lingual frenulum evaluation protocol with baby scores^(15)^, where only infants with scores of seven or greater were considered to have ankyloglossia. After the clinical evaluation, the infant was offered a silicone pacifier (Soothie, Philips Avent.) connected to the pressure acquisition device. Non-nutritive suction was evaluated for 120 seconds, with the baby on the mother’s lap in the breastfeeding position and without having eaten for a period of 1 hour.

### Suction recording device

**Figure 1:**
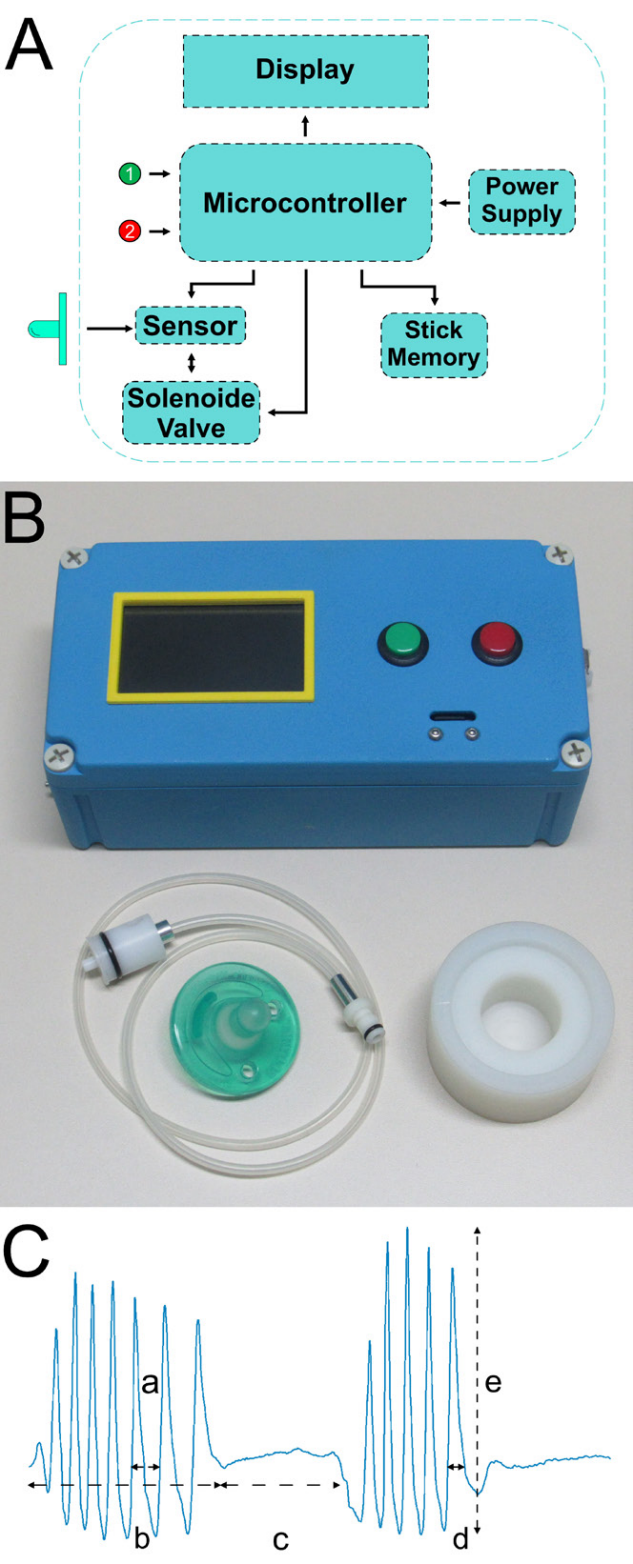
Representation of the prototype, signal and extracted variables. **A**. Block diagram of the prototype. The pressure sensor is connected to one of the microcontroller A/D ports on the platform. The color display shows the profile of the pressure variations in the pacifier graphically. The data relating to pressure sweeps is stored sequentially in a memory card connected to the platform. The pacifier was connected to the sensor through a silicone tube. A stainless-steel tube was introduced to allow the air to flow between the pacifier and the pressure sensor. The solenoid valve connects the pressure measurement route with the external environment. **B**. Front view of the device with pacifier and the connection. **C**. Representative recording of non-nutritive suction activity from an infant, and an explanation of the analyzed variables. Note that sucks cluster in stereotypical bursts of activity. a: sucks per burst. b: burst width c: inter-burst interval. d: suck duration. e: maximum amplitude of sucks per burst

The device uses a prototyping platform (Due, Arduino.) to capture the silicone pacifier's pressure variations. The signals from a pressure sensor (MPS20N0040D-S, e-Radionica.com) connected to one of the microcontroller A/D ports on the platform were digitized at 12 bits with a sample rate of approximately 10 kHz (Figure 1). A data acquisition program was developed in JAVA and shipped to record the pressure variations and show the profile of these pressure variations in the pacifier graphically through a color display (LCD TFT Touch 3.5″). The pressure due to the pacifier compression is automatically compensated through a solenoid valve that connects the pressure measurement route with the external environment. By doing so, it is possible to correct spurious pressure variations that could contaminate data acquisition.

The data relating to pressure sweeps were stored sequentially in a memory card (8 GB M2 Sandisk, Western Digital Technologies) connected to the platform.

The pacifier was connected to the sensor through a 1.5 m silicone tube with 4 mm outside diameter and 2.5 mm inner diameter. To connect the pacifier to the tube, a Teflon ™ cylinder was constructed that perfectly fits the pacifier's opening. A stainless steel tube was introduced to allow the air to flow between the pacifier and the pressure sensor. To avoid accidental contacts between the researcher and the pacifier, a Nylon ™ holder was specially designed to support the pacifier during the exchange process, as shown in Figure 1. All data collected during the registration phase were stored in a hard drive for further offline analysis.

### Data analysis

Butterworth's low-pass filter was applied to the recorded signals in order to reduce noise and preserve signal characteristics. The signal was divided into bursts, defined as groups of sucks with a minimum duration of 0.035 seconds and a minimum of 3 suctions (maximum time of 1 second between pulses), which occurred at close time intervals. Thus, we extracted the frequency of sucks within a burst, the average suck duration (time differences between sucks), the burst duration (bursts between intervals greater than 1.5 seconds), the number of sucks per burst (determined by the temporal and maximum values of the sucks, where the maximum values are followed and preceded by short drops because intervals longer than 1,5 seconds without action are considered the beginning and end of the bursts), the maximum amplitude of sucks per burst (determined by the same methodology as the number of sucks per burst) and the inter-burst interval (interval between bursts lasting longer than 1.5 seconds)^(16)^ (Figure 1). To complete the analysis of the data obtained, the root mean square (RMS) was calculated in the parameter number of sucks per burst, in order to test the magnitude of the signal and reach average power. These variables were selected because we reasoned that frequency and intensity of suction behavior would likely be the most altered parameters in the infants of ankyloglossia, and these measures should provide a broad description of these two main effects.

### Statistical analysis

Most parameters were found to be non-normally distributed using the Shapiro-Wilk normality test, and therefore the unpaired t-test was used for comparisons between groups. Significance level was set at 0.05 for all analyses.

### Data availability

The datasets generated during and/or analyzed during the current study are available from the corresponding author on reasonable request.

## RESULTS

### Sample characterization

The sample of this research is composed of 55 babies, of which 36 make up the control group and 19 the ankyloglossia group. The control group contained 17 males and the ankyloglossia group had 16 males. The average age for controls was 2.51 days (± 1,39) and 5.35 days (± 7,96) in the group with ankyloglossia. In both groups, all babies were born at full-term and had no complications during delivery and/or complications after birth. One of the inclusion criteria was to be breastfed, but 16 (45%) babies in the control group were supplementing with formula compared to only 4 (21%) in the ankyloglossia group, even though the mothers of subjects in the latter group frequently complained of pain when breastfeed (64%). We observed a higher frequency of reports of long feedings in the group with ankyloglossia (43%), compared to only 16% of mothers in the control group (Table 1).

**Table 1:** Demographics and breastfeeding characteristics of babies

	**CONTROL**	**ANKYLOGLOSSIA**
Total	36	19
**Sex**		
Male	17	16
Female	19	03
**Age (days, mean±SD)**	2.51 ± 1.39	5.35 ± 7.96
**Formula supplementation (%)**	16 (45%)	4 (21%)
**Reported pain**	8 (22%)	12 (64%)
**Long feedings**	6 (16%)	8 (43%)

### Recording suction activity from newborns

**Figure 2:**
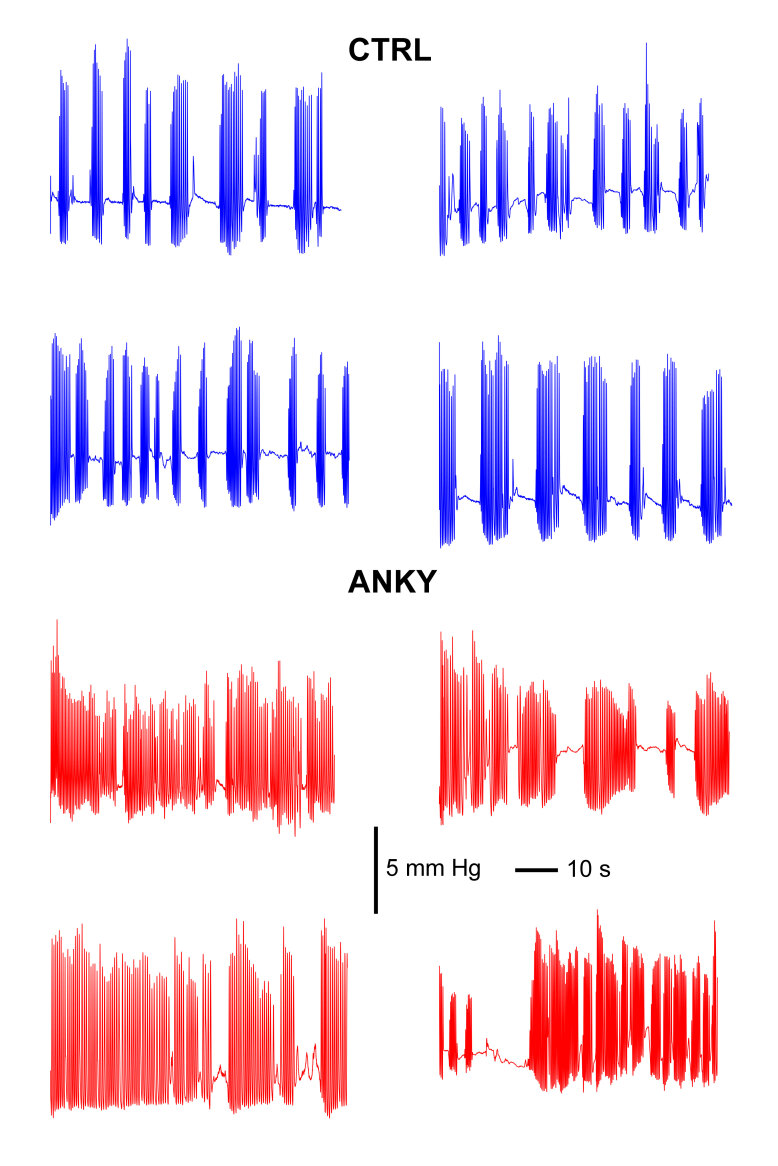
Representative recordings from four subjects from the control (blue) and ankyloglossia (red) groups. Note that babies diagnosed with ankyloglossia have clearly longer bursts in comparison to controls. Scale bars are applicable to all recordings

We first developed a novel inexpensive device for measuring suction dynamics in infants, which is essentially a microprocessor-controlled pressure sensor coupled to a silicone pacifier (Figure 1). We used this device to record non-nutritive suction activity from 19 babies with ankyloglossia (ANKY) and 36 control babies (CTRL). Our device allowed for the high resolution recording of pressure changes within the pacifier, which were characterized by stereotypical “bursts” of suctions (Figures 1C and 2). We extracted from these recordings the following variables: frequency of sucks within a suction burst, suction burst duration, the number of sucks per burst, the duration of individual sucks, the maximum amplitude of sucks per burst and the inter-burst interval (Figure 1).

### Ankyloglossia impacts suction patterns

**Figure 3:**
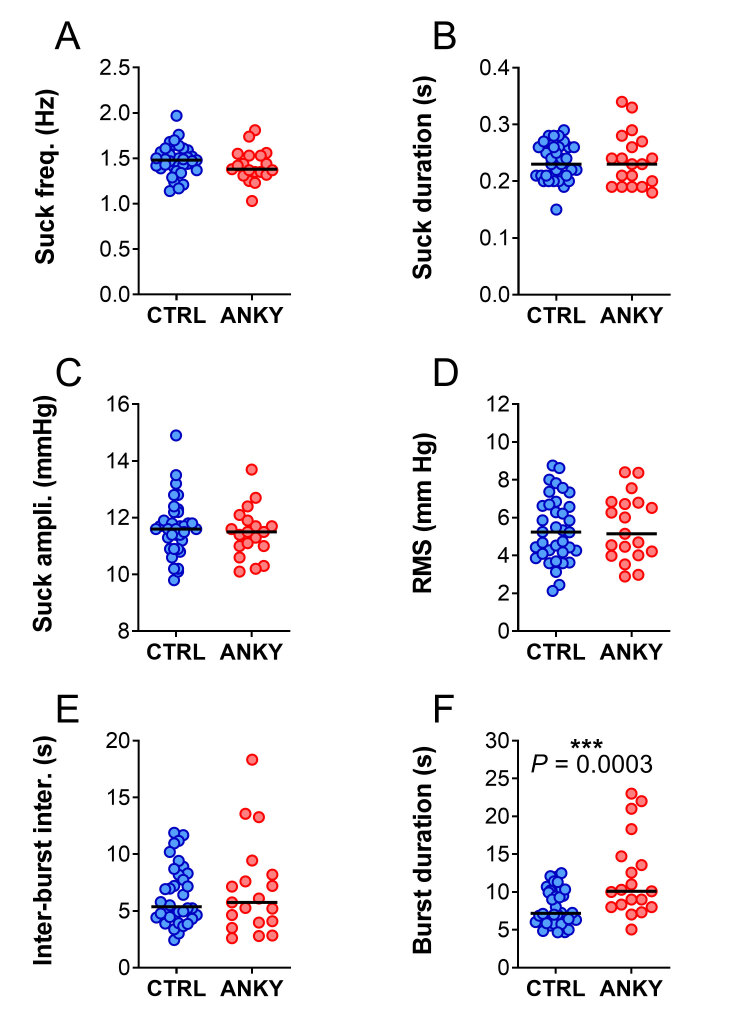
Objective measures of sucking patterns in the control (blue) and ankyloglossia (red) groups. A: mean frequency of sucks. B: mean duration of individual sucks. C: mean amplitude of sucks per burst. D: mean RMS of number of sucks per burst. E: mean inter-burst interval greater than 1.5 seconds. E. mean burst between intervals greater than 1.5 seconds, possible to note a significant increase in the ankyloglossia’s group. Black lines indicate medians. F. mean duration of bursts

Infants with ankyloglossia showed strikingly different suction activity patterns compared to controls (Figure 2). Nevertheless, most objective parameters that we analyzed were similar between groups. This included mean suck frequency (Figure 3A, p= 0.2210), the mean duration of each suck (Figure 3B, p= 0.7292), the amplitude of sucks (Figure 3C, p= 0.5586), signal root-mean-square (RMS, Figure 3D, p=0.8428) and inter-burst interval (Figure 3E, p= 0.9898). The only parameter that was clearly different was the mean suction burst duration (Figure 3F, p= 0.0003), indicating that babies with ankyloglossia suck more often and for longer periods of time.

### Influence of ankyloglossia on maternal complaints.

**Figure 4:**
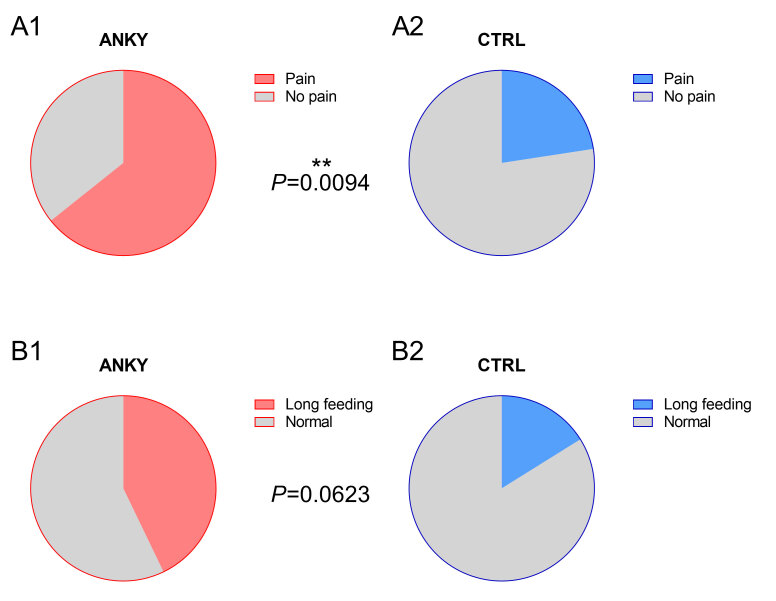
Subjective measures of pain during breastfeeding and long feeding in the control (blue) and ankyloglossia (red) groups. Comparison of the presence of pain during breastfeeding in both groups revealed a significant increase in the group with ankyloglossia (upper panels, p<0.01) and a trend increase for reports of longer feeding the group with ankyloglossia

With the data collected in the anamnesis, we found that mothers of infants in the ankyloglossia’s group reported significantly more pain complaints (64% versus 22%, p= 0.0094), and there was a strong trend towards more reported long feedings (43% versus 16%, p=0.0623). These findings are shown in Figure 4.

## DISCUSSION

We developed a novel method for monitoring non-nutritive sucking in infants using a custom-built pressure-meter coupled to a pacifier. We tested this approach on newborn infants with and without ankyloglossia, or tongue-tie, a congenital anomaly known to impact breastfeeding^(17,18)^. We found that subjects with ankyloglossia performed longer suction bursts, while most other suction parameters were similar to controls.

There are two different types of infant suction: nutritive (NS) and non-nutritive (NNS). The first is when milk or any other liquid is ingested, like in typical breastfeeding, and the second is when there is no fluid intake, such as when the infant is using a pacifier^(19)^. These differences in types of sucking are correlated differences in tongue movements. In NS, the tongue lowers to create an intraoral vacuum and accommodates breast milk, and sucking is significantly slower when NNS is compared. In the latter, the tongue does not need to go down in the same proportion^(20)^. This has been confirmed by other studies that claim that the baby's jaw movements are shorter and faster during NNS^(21)^. Thus, it is clear that the presence of milk alters tongue movements. Despite these known differences, we reasoned that objectively measuring NNS with our device could be a useful indicator of NS development during breastfeeding. Indeed, there is previous evidence that NNS matures earlier, this is because it doesn’t need to be coordinated with swallowing and breathing because it does not have liquid intake (besides saliva), and its stimulation positively influences on the improvement of the NS pattern^(9,19,22)^. Given that the presence of milk during sucking modifies tongue movements, we believe that this was one of the reasons for not detecting differences in the other evaluated parameters. Therefore, as pressure variation records were not performed concomitantly with breastfeeding, but using a pacifier, variables that could contribute to variation in other parameters were not computed, such as, for example, the swelling of the maternal breast in depending on the amount of milk and nipple size. Thereby, the only parameter that showed a difference, using this methodology, is not related to the others that could certainly present variation if the measurements were conducted directly in the mother's breast. That said, our method may not reveal potential differences in nutritive suction that are due to sensorimotor feedback during fluid intake and swallowing. We believe our approach may be more adequate for evaluating structural limitations in sucking capacity, such as those caused by ankyloglossia.

Infants with ankyloglossia ingest less milk in each breastfeeding session, and therefore need to breastfeed more times a day^(23)^. It is believed that infants with ankyloglossia need to breastfeed longer to compensate for inefficient feeding, and the longer time at the breast may contribute to the pain reported by the mothers. Our findings are in line with this interpretation, as we found that infants with ankyloglossia had an 66.6% increase in the burst duration, which paralleled more complaints of pain when breastfeeding from their mothers. This would mean that not only do these infants have longer feeding sessions, but that the pattern of suction within each section is also biased towards longer attempts at feeding. It is likely that these two effects are additive and contribute to the pain and discomfort experienced by lactating mothers.

We believe there could be several advantages of using our device for screening newborn suction patterns. Due to its low cost and simple signal output, it would be relatively simple to scale its application to several units in a maternity ward. Through it, a healthcare provider could potentially detect deficits in suction capacity at an early stage. In clinical practice, early diagnosis and effective interventions by a lactation expert could avoid maternal pain and perhaps even early weaning of babies with ankyloglossia. Going further, the device can also be used as a therapeutic monitoring resource, where pre- and post-intervention comparisons can be made in order to support approaches, such as frenectomy or, in different cases, the release of oral feeding of babies using an orogastric tube. Of course, future studies would be needed to validate these suggestions, and to establish a more solid link between the monitoring of non-nutritive suction, maternal pain, and breastfeeding capacity at the individual level.

A major limitation of our study was the small number of participants with ankyloglossia, which precluded us from attempting to segregate the data from babies with ankyloglossia into groups according to the type or severity of the malformation. Future work should address whether specific differences across different manifestations of ankyloglossia can also be revealed using the approach described here.

## CONCLUSION

In conclusion, we provide here a new, inexpensive, and relatively simple method for obtaining direct recordings of infant non-nutritive suction. This method can assist with the severity evaluation of ankyloglossia, which we found to affect primarily the duration of suction bursts, which is likely linked to the characteristic suboptimal and pain-inducing feeding patterns of infants with ankyloglossia. Given the public health impact of early weaning^(24)^ and the use of frenectomy, further studies should be done to fully characterize the relationship between functional changes in suction capacity and actual breastfeeding efficiency, as well as to quantify the actual benefits of surgical interventions.
